# Supplementary material for: Novel Driver Strength Index highlights important cancer genes in TCGA PanCanAtlas patients
Source: PeerJ. 2022 Aug 11;10:e13860. doi: 10.7717/peerj.13860 (PMC9375969; doi:10.7717/peerj.13860)
Supplement: Supplemental Information 2 [file peerj-10-13860-s002.zip › COHORTS/genes plots/cohorts/2021_8_16_14_9_distribution_cohorts_SARC.pdf]

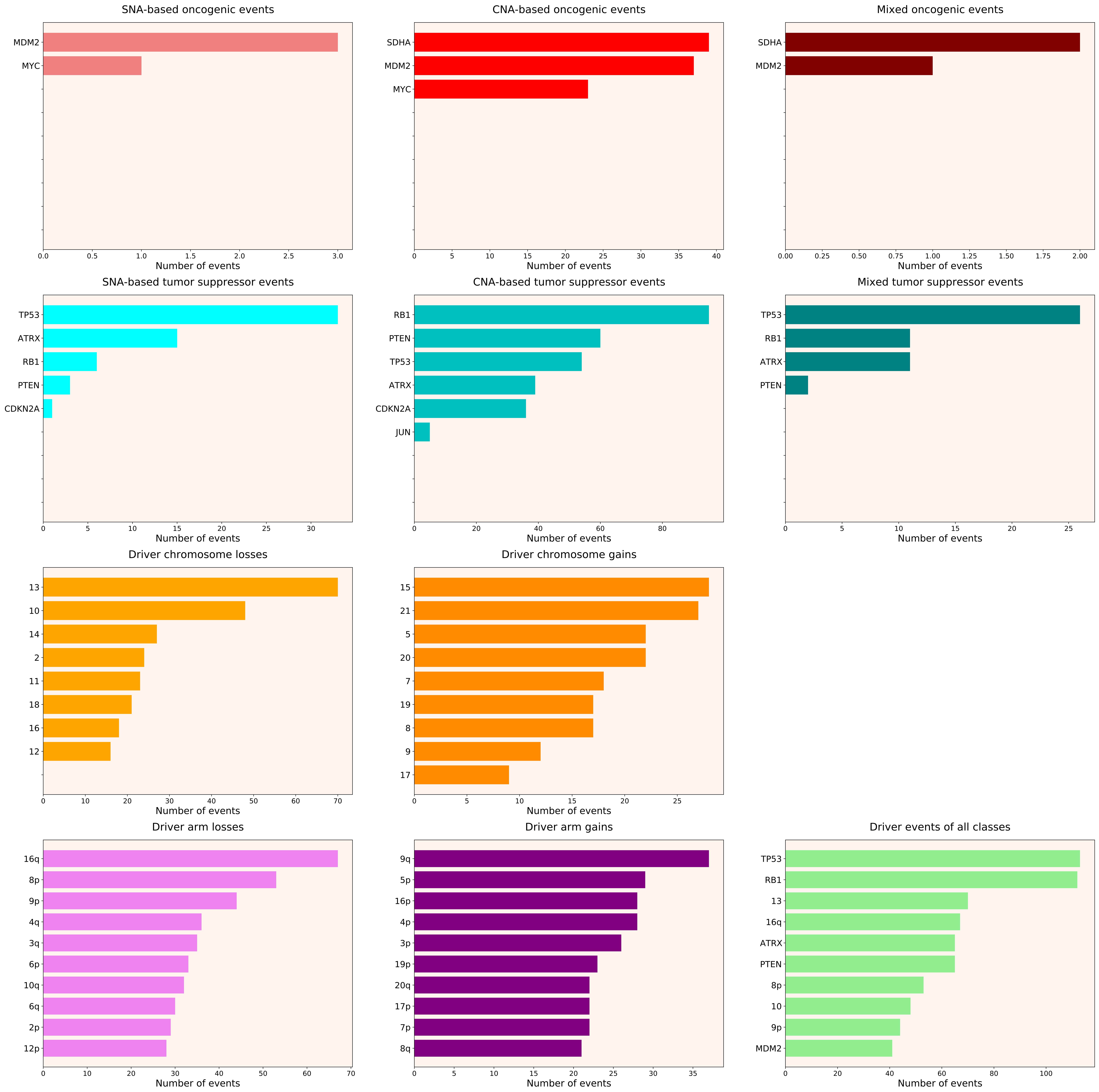
SNA-based oncogenic events
MDM2
MYC
0.0 0.5 1.0 1.5 2.0 2.5 3.0
Number of events
CNA-based oncogenic events
SDHA
MDM2
MYC
0 5 10 15 20 25 30 35 40
Number of events
Mixed oncogenic events
SDHA
MDM2
0.00 0.25 0.50 0.75 1.00 1.25 1.50 1.75 2.00
Number of events
SNA-based tumor suppressor events
TP53
ATRX
RB1
PTEN
CDKN2A
0 5 10 15 20 25 30
Number of events
CNA-based tumor suppressor events
RB1
PTEN
TP53
ATRX
CDKN2A
JUN
0 20 40 60 80
Number of events
Mixed tumor suppressor events
TP53
RB1
ATRX
PTEN
0 5 10 15 20 25
Number of events
Driver chromosome losses
13
10
14
2
11
18
16
12
0 10 20 30 40 50 60 70
Number of events
Driver chromosome gains
15
21
5
20
7
19
8
9
17
0 5 10 15 20 25
Number of events
Driver arm losses
16q
8p
9p
4q
3q
6p
10q
6q
2p
12p
0 10 20 30 40 50 60 70
Number of events
Driver arm gains
9q
5p
16p
4p
3p
19p
20q
17p
7p
8q
0 5 10 15 20 25 30 35
Number of events
Driver events of all classes
TP53
RB1
13
16q
ATRX
PTEN
8p
10
9p
MDM2
0 20 40 60 80 100
Number of events
